# Mapping of etiologies of computed tomography-proven acute colitis: a prospective cohort study

**DOI:** 10.1038/s41598-022-13868-w

**Published:** 2022-06-13

**Authors:** Jeremy Meyer, Jacques Schrenzel, Alexandre Balaphas, Vaihere Delaune, Mohamed Abbas, Philippe Morel, Giaccomo Puppa, Laura Rubbia-Brandt, Philippe Bichard, Jean-Louis Frossard, Christian Toso, Nicolas C. Buchs, Frédéric Ris

**Affiliations:** 1 Division of Digestive Surgery, University Hospitals of Geneva, Rue Gabrielle-Perret-Gentil 4, 1211 Geneva 14, Switzerland; 2 Section of Clinical Medicine, Medical School, University of Geneva, Rue Michel-Servet 1, 1206 Geneva, Switzerland; 3 Laboratory of Bacteriology, University Hospitals of Geneva, Rue Gabrielle-Perret-Gentil 4, 1211 Geneva 14, Switzerland; 4 Division of Infectious Diseases, University Hospitals of Geneva, Rue Gabrielle-Perret-Gentil 4, 1211 Geneva 14, Switzerland; 5 Department of Pathology, University Hospitals of Geneva, Rue Gabrielle-Perret-Gentil 4, 1211 Geneva 14, Switzerland; 6 Division of Gastroenterology, University Hospitals of Geneva, Rue Gabrielle-Perret-Gentil 4, 1211 Geneva 14, Switzerland; 7 Département de chirurgie, Clinique Générale-Beaulieu, Genève, Switzerland

**Keywords:** Medical research, Epidemiology, Outcomes research, Translational research

## Abstract

Our objective was to describe the etiologies of acute colitis and to identify patients who require diagnostic endoscopy. Patients with symptoms of gastrointestinal infection and colonic inflammation on CT were prospectively included. Those immunosuppressed, with history of colorectal cancer or inflammatory bowel disease (IBD), were excluded. Microbiological analysis of the feces was performed using PCR assays BD-Max and FilmArray (GI panel,) and fecal cultures. Fecal calprotectin was determined. Patients with negative BD-Max underwent colonoscopy. One hundred and seventy-nine patients were included. BD-Max was positive in 93 patients (52%) and FilmArray in 108 patients (60.3%). Patients with infectious colitis (n = 103, 57.5%) were positive for *Campylobacter* spp. (n = 57, 55.3%), *Escherichia coli* spp. (n = 8, 7.8%), *Clostridioides difficile* (n = 23, 22.3%), *Salmonella* spp. (n = 9, 8.7%), viruses (n = 7, 6.8%), *Shigella* spp. (n = 6, 5.8%), *Entamoeba histolytica* (n = 2, 1.9%) and others (n = 4, 3.9%). Eighty-six patients underwent colonoscopy, which was compatible with ischemic colitis in 18 patients (10.1%) and IBD in 4 patients (2.2%). Fecal calprotectin was elevated in all patients, with a mean concentration of 1922.1 ± 2895.6 μg/g, and was the highest in patients with IBD (8511 ± 9438 μg/g, *p* < 0.001). After exclusion of patients with infectious etiology, a fecal calprotectin > 625 μg/g allowed identifying patients with IBD with an area under ROC curve of 85.1%. To conclude, computed tomography-proven colitis was of infectious etiology in 57.5% of patients. The main pathogens identified were *Campylobacter* spp. (55.3%), *Clostridioides difficile* (22.3%) and *Salmonella* spp. (8.7%). Ischemic colitis (10.1%) and IBD (2.2%) were seldom represented. No colorectal cancer was found.

## Introduction

The term "colitis" etymologically refers to the inflammation of a segment of the colon. The inflammation can be confirmed by endoscopy^1^, histology^2,3^ or radiology, the latest showing a thickening of the colon wall and infiltration of the surrounding fat not explained by an alternative diagnosis^4^. Furthermore, for diagnosing colitis, the inflammation of the colon must be symptomatic, in order to distinguish an episode of colitis from aasymptomatic thickening of the colon fortuitously discovered on computed tomography (CT), which may have alternative aetiologies^5,6^.

Classification is usually made into infectious colitis, inflammatory bowel disease (IBD), ischemic colitis and iatrogenic colitis (notably due to non-steroidal anti-inflammatory drugs (NSAID))^7^. The aetiologies of colitis affect therapeutic choices, since patients with infectious colitis might improve spontaneously or with appropriate antibiotics, those with IBD will require the introduction of immunosuppressive therapy and follow-up, and those with ischemic colitis will need supportive treatment and careful evaluation to detect any progression to bowel perforation which would prompt for emergency surgery.

Due to the increasing use of CT in case of abdominal symptoms in the emergency setting, visualization of an inflamed segment of colon, not explained by an alternative diagnosis such as diverticulitis, is nowadays encountered on a daily basis by emergency, gastroenterology and general surgery teams. However, the literature does not provide any recommendation regarding the diagnostic management to perform in patients with CT-proven episode of colitis. Moreover, the prevalence of the different possible aetiologies of colitis remains unknown and feces of these patients have—so far—not been screened using multiarray PCR assays. As a consequence, the difficulty encountered in the early identification of patients requiring endoscopy for suspected IBD or cancer may delay or even contribute to missing these diagnoses, especially if the acute phase of IBD has passed^8^.

Therefore, considering the absence of scientific evidence in the field and in order to improve the management of patients with acute colitis, we aimed to: (1) describe the clinical and para-clinical presentations of patients with acute colitis, defined as a symptomatic disease associated with a CT-compatible picture, (2) determine the etiologies of acute colitis, and (3) identify the subgroup of patients requiring diagnostic colonoscopy to exclude IBD (the PICO question is summarized in Table S1).

## Materials and methods

### Registration

This monocentric prospective cohort study was reviewed and approved by the local ethics committee of Geneva, Switzerland, and registered into clinicaltrials.gov (NCT02709213). The study complied with the STROBE statement (Table S2). All methods were performed in accordance with relevant guidelines and regulations.

### Patient and public involvement

Patients or the public were not involved in the design, or conduct, or reporting, or dissemination plans of our research.

### Data accessibility

The data underlying this article will be shared on reasonable request to the corresponding author.

### Study design

The study was a monocenter prospective cohort study conducted in a tertiary center. The study design is reported in Fig. S1.

### Study population

Patients presenting at the emergency department of the University Hospitals of Geneva, Switzerland, with one or more symptom(s) compatible with gastrointestinal infection (fever (central temperature ≥ 38 °C) and/or acute abdominal pain and/or diarrhea) and signs of colonic inflammation on CT (as reported previously^4^) were considered having an episode of acute colitis and as eligible for inclusion. Informed written consent to participate was sought. Patients with inflammation centered around a diverticulum and/or with suspected diverticulitis were not considered for inclusion. Patients unable to give informed consent, those with a personal history for IBD and/or colorectal cancer, those taking immunosuppressive medication and/or immunosuppressed, those with abdominal ascites due to liver cirrhosis, those who could not proceed to follow-up by the Swiss IBD cohort (foreign residents) and those refusing to participate, were excluded. Failure to perform the required examinations (molecular panels and/or colonoscopy) was considered as violation of study protocol (drop-out).

### Management of patients with computed tomography-proven acute colitis

Patients suffering from CT-proven acute colitis and compatible symptoms were routinely hospitalized. Blood tests were collected at admission and then according to clinical judgement. First feces were collected in Cary-Blair medium and sent for microbiological analyses using BD-Max. Additional fecal samples were collected and stored in a 4 °C fridge for a maximum of 24 h, before being transferred to a − 80 °C freezer. These samples were sent to an external laboratory (Unilabs, Geneva, Switzerland) for calprotectin determination. Additional microbiological analyses were performed in parallel using FilmArray GI panel in all patients. Antibiotic treatment with intravenous 2g ceftriaxone 1×/day and oral 500mg metronidazole 3×/day was initiated from admission and continued for at least 5 days, according to institutional guidelines. Then, antibiotics treatment was continued *per os* using 500mg ciprofloxacine 2×/day and 500mg metronidazole 3×/day for a total of 10 days in case of episode of undetermined etiology, or adapted to microbiological results from the fecal samples.

### Microbiological analyses

Routine microbiological analyses included PCR using BD-Max panel (BD-Max TM Enteric Bacterial panel) detecting *Campylobacter* spp*.*,* Salmonella* spp., *Shigella* spp.,* Shiga* toxins 1&2, *Enterohaemorragic Escherichia coli (EHEC)* and *Enteroinvasive Escherichia coli (EIEC*). If requested by the ordering physician, PCR for *Clostridioides difficile* (BD-Max™ *C. difficile* detecting the toxin B gene) and *Yersinia* spp. were added. If BD-Max was positive, bacterial pathogens were systematically checked for by appropriate fecal culture. In parallel, all fecal samples were analyzed in-house by the BioFire FilmArray GI panel (bioMérieux, Salt Lake City, USA). This assay allows detection of 22 gastro-intestinal pathogens, including *Campylobacter jejuni/coli/upsaliensis, Clostridioides difficile (toxins A/B), Plesiomonas shigelloides, Salmonella* spp.,* Yersinia enterocolitica, Vibrio parahaemolyticus/vulnificus/cholerae, Enteroaggregative Escherichia coli (EAEC), Enteropathogenic Escherichia coli (EPEC), Enterotoxigenic Escherichia coli (ETEC) lt/st, Shiga-like toxin-producing Escherichia coli (STEC) stx1/stx2, Shigella/EIEC, Cryptosporidium* spp.,* Cyclospora cayetanensis, Entamoeba histolytica, Giardia lamblia, Adenovirus F40/41, Astrovirus, Norovirus GI/GII, Rotavirus A* and *Sapovirus I/II/IV/V*.

### Cross-matching with local databases for inflammatory bowel disease

The cohort was matched with the Swiss IBD Cohort Study on the 28.04.2020 to check for potential additional cases of IBD.

### Colonoscopy

If BD-Max yielded negative results, colonoscopy was performed during the hospital stay. Patients were hospitalized until positive BD-Max was obtained or, if negative, until colonoscopy was performed. An experienced gastroenterologist performed colonoscopy. Standard split-dose bowel preparation with polyethylene glycol was used. Intravenous sedation was performed with propofol. Colonoscopies were performed with high-definition 190 series CF or PCF (Olympus, Tokyo, Japan) using CO_2_ insufflation. In case of appearance of mucosal inflammation, images were taken and integrated in the endoscopy report, biopsies were taken and sent for histological analysis. Polyps, if found, were removed and sent for histological analysis.

### Definition of the etiologies of acute colitis

The episode of acute colitis was considered as infectious if either the routine BD-Max or the multiarray PCR assay FilmArray GI panel was positive. *Escherichia coli* spp. EAEC, EPEC and ETEC identified only by FilmArray GI panel were considered as carriage due to ongoing doubts regarding their pathogenicity. Pathogens were further identified by fecal culture. If BD-Max was negative, diagnosis was obtained using colonoscopy with or without biopsies. In non-infected cases, we defined the etiology of colitis as ischemic, or secondary to colorectal cancer or IBD in the presence of suggestive macroscopic aspect and/or histopathology. Patients later registered in the Swiss IBD cohort were considered to have IBD. To avoid missing a secondary diagnosis in patients with infectious etiology, medical files were reviewed at the date of the inclusion of the last patient to rule out later diagnosis of IBD and/or colorectal cancer. Undetermined colitis, in which the etiology of acute colitis was unknown, was defined as normal colonoscopy and, if available, normal histology.

### Statistical analysis

All statistical analyses were performed using STATA (version 13, StataCorp LP, College Station, USA) and GraphPad PRISM (version 7, GraphPad Software, La Jolla California USA). The null hypothesis was rejected at *p* < 0.05. Network representation of pathogens identified using the FilmArray GI panel was performed using Cytoscape (version 3.8.1).

#### Differences between groups

Differences between groups (etiologies of colitis) were determined using the Student’s two-sided t-test or the Pearson’s chi-squared test, as appropriate. Continuous variables were transformed into categorical variables if required. Variables were expressed as proportions for categorical variables and means for continuous variables; 95% confidence intervals (95% CI) and standard deviations (SD) were reported, as appropriate.

#### Receiver operating characteristic (ROC) curve

To define the optimal cut-off point of fecal calprotectin allowing identifying patients suffering from IBD, a ROC curve was drawn. Standard error was calculated according to the DeLong method. The optimal cut-off point was determined using the Liu method.

## Results

### Inclusion process

From December 2016 to December 2019 (3 years), 300 patients were potentially eligible for inclusion in the study. Sixty-two patients (20.7%) were excluded for fulfilling at least one exclusion criteria (history of IBD: 14 patients, immunosuppression: 14 patients, inability to provide informed consent: 10 patients, no possibility for follow-up (foreign resident): 10 patients, liver cirrhosis with ascites: 8 patients, history of colorectal cancer: 4 patients, refuse to participate: 2 patients), leaving 238 patients for inclusion. Among those, biological samples were unavailable (improperly harvested or stored) in 33 patients, colonoscopy was not performed during hospitalization or was postponed after the acute phase in 18 patients, and 8 patients refused colonoscopy. Therefore, 59 patients (representing 24.8% drop-outs) were further excluded, leaving 179 patients for final analysis (Fig. S2).

### Patient demographics

Included patients had a mean age of 53.1 ± 20.6 years and 71 (39.7%) were males. Eighty-one (45.3%) had at least one cardiovascular risk factor. Fifteen (8.4%) experienced a previous episode of acute colitis (of non-IBD etiology). Twenty-four (13.4%) used NSAID within the last 2 weeks and 29 (16.2%) received antibiotics within the last 4 weeks (Table 1).

**Table 1:** Characteristics of included patients.

	All patients, *n* = 179	Infectious colitis, *n* = 103	*p* value*	Ischemic colitis, *n* = 18	*p-* value*	Inflammatory bowel disease *n* = 4	*p-* value*	Undetermined, *n* = 54	*p-* value*
**Demographics**									
Age, year, mean ± SEM	53.1 ± 20.6	49.2 ± 19.7	*0.023*	72 ± 12.4	< *0.001*	25 ± 4.7	*0.005*	56.5 ± 20	*0.147*
Gender, male (%)	71 (40%)	45 (43.7%)	*0.200*	5 (27.8%)	*0.277*	1 (25%)	*0.544*	20 (37%)	*0.637*
Cardiovascular risk factor(s), yes (%)	81 (45.3%)	40 (38.8%)	*0.045*	13 (72.2%)	*0.015*	1 (25%)	*0.411*	27 (50%)	*0.402*
Hypertension, yes (%)	50 (27.9%)	20 (19.4%)	–	12 (66.7%)	–	0 (0%)	–	18 (33.3%)	–
Dyslipidemia, yes (%)	28 (15.6%)	13 (12.6%)	–	5 (37.8%)	–	0 (0%)	–	10 (18.5%)	–
Diabetes, yes (%)	21 (11.7%)	10 (9.7%)	–	5 (27.8%)	–	0 (0%)	–	6 (11.1%)	–
Tobacco use, yes (%)	20 (11.2%)	11 (10.7%)	–	2 (11.1%)	–	1 (25%)	–	6 (11.1%)	–
History of colitis, yes (%)	15 (8.4%)	9 (8.7%)	*0.841*	1 (5.6%)	*0.648*	0 (0%)	*0.541*	5 (9.3%)	*0.780*
Recent NSAID use, yes (%)	24 (13.4%)	13 (12.6%)	*0.719*	4 (22.2%)	*0.247*	1 (25%)	*0.491*	6 (11.1%)	*0.553*
Recent antibiotherapy, yes (%)	29 (16.2%)	19 (18.5%)	*0.343*	2 (11.1%)	*0.537*	3 (75%)	*0.001*	5 (9.3%)	*0.098*
**Anamnesis**									
Abdominal pain, yes (%)	169 (94.4%)	97 (94.2%)	*0.871*	17 (94.4%)	*0.995*	4 (100%)	*0.623*	51 (94.4%)	*0.991*
Fever, yes (%)	92 (51.4%)	70 (68%)	< *0.001*	5 (27.8%)	*0.035*	2 (50%)	*0.955*	15 (27.8%)	< *0.001*
Diarrhea, yes (%)	147 (82.1%)	99 (96.1%)	< *0.001*	12 (66.7%)	*0.071*	2 (50%)	*0.090*	34 (63%)	< *0.001*
**Clinical examination**									
Temperature (°C) mean ± SD	37.3 ± 0.9	37.5 ± 0.9	< *0.001*	37.1 ± 0.7	*0.372*	37.1 ± 0.6	*0.760*	37 ± 0.7	*0.001*
Temperature ≥ 38 °C, yes (%)	35 (19.8%)	27 (26.2%)	*0.011*	3 (16.7%)	*0.727*	4 (100%)	*0.315*	5 (9.6%)	*0.029*
Heart frequency (bpm/min), mean ± SD	87.7 ± 16.7	92 ± 16.3	< *0.001*	87.4 ± 17.8	*0.943*	82.5 ± 8.6	*0.530*	79.9 ± 15	< *0.001*
Heart frequency ≥ 100 bpm/min, yes (%)	46 (25.7%)	36 (35%)	*0.001*	2 (22.2%)	*0.722*	0 (0%)	*0.234*	6 (11.1%)	*0.003*
Systolic blood pressure (mmHg), mean ± SD	124.7 ± 17.6	121.4 ± 15.1	*0.003*	133.4 ± 19	*0.025*	112.3 ± 5.1	*0.153*	129 ± 20	*0.031*
Systolic blood pressure < 100 mmHg, yes (%)	8 (4.5%)	5 (4.9%)	*0.772*	1 (5.6%)	*0.814*	0 (0%)	*0.662*	2 (3.7%)	*0.745*
Abdominal pain, yes (%)									
Present	167 (93.3%)	97 (94.2%)	*0.584*	17 (94.4%)	*0.837*	4 (100%)	*0.588*	49 (90.7%)	*0.369*
Diffuse	26 (14.5%)	15 (14.6%)	*0.987*	6 (33.3%)	*0.017*	1 (25%)	*0.548*	4 (7.4%)	*0.076*
Abdominal tenderness, yes (%)									
Present	44 (24.6%)	26 (25.2%)	*0.451*	5 (27.8%)	*0.853*	1 (25%)	*0.977*	12 (22.2%)	*0.559*
Diffuse	1 (0.6%)	1 (1%)	–	0 (0%)	–	0 (0%)	–	0 (0%)	–
Abdominal rebound pain, yes (%)									
Present	26 (14.5%)	15 (14.6%)	*0.473*	2 (11.1%)	*0.805*	3 (75%)	*0.002*	6 (11.1%)	*0.433*
Diffuse	0 (0%)	0 (0%)	–	0 (0%)	–	0 (0%)	–	0 (0%)	–
**Paraclinical examination**									
Blood tests									
Hemoglobin (g/l), mean ± SD	136.4 ± 18.1	137.3 ± 17.5	*0.414*	136.7 ± 19.6	*0.946*	128.8 ± 20.6	*0.391*	135.1 ± 18.8	*0.517*
Hemoglobin < 100 g/l, yes (%)	4 (2.2%)	1 (1%)	*0.183*	0 (0%)	*0.499*	1 (25%)	*0.002*	2 (3.7%)	*0.382*
Leukocytes (G/l), mean ± SD	10.6 ± 4.1	9.8 ± 3.6	*0.003*	11.4 ± 4.9	*0.363*	12.3 ± 3.7	*0.405*	11.7 ± 4.6	*0.021*
Leukocytes ≥ 12G/l, yes (%)	62 (34.6%)	27 (26.2%)	*0.006*	5 (27.8%)	*0.519*	3 (75%)	*0.002*	27 (50%)	*0.005*
CRP (mg/l), mean ± SD	90.4 ± 89.3	113.1 ± 90.4	< *0.001*	58.1 ± 97.1	*0.106*	85.1 ± 74.1	*0.905*	58.2 ± 73	*0.001*
CRP ≥ 50 mg/l, yes (%)	105 (58.7%)	76 (73.8%)	< *0.001*	6 (33.3%)	*0.021*	3 (75%)	*0.502*	20 (37%)	< *0.001*
CRP ≥ 200 mg/l, yes (%)	22 (12.3%)	17 (16.5%)	*0.046*	1 (5.6%)	*0.359*	0 (0%)	*0.449*	4 (7.4%)	*0.191*
Calprotectin (μg/g), mean ± SD	1922.1 ± 2895.6	2194.3 ± 2764.2	*0.158*	1357.2 ± 1011.1	*0.383*	8511.4 ± 9438.4	< *0.001*	1113.5 ± 1936.5	*0.014*
Calprotectin ≥ 250 μg/g, yes (%)	132 (73.7%)	86 (83.5%)	*0.001*	15 (83.3%)	*0.330*	4 (100%)	*0.227*	27 (50%)	< *0.001*
Calprotectin ≥ 2500 μg/g, yes (%)	40 (22.4%)	28 (27.2%)	*0.070*	3 (16.7%)	*0.542*	2 (50%)	*0.179*	7 (13%)	*0.048*
Calprotectin ≥ 5000 μg/g, yes (%)	29 (16.2%)	23 (22.3%)	*0.010*	0 (0%)	*0.049*	2 (50%)	*0.064*	4 (7.41%)	*0.036*
Calprotectin ≥ 7500 μg/g, yes (%)	17 (9.5%)	13 (12.6%)	*0.097*	0 (0%)	*0.147*	2 (50%)	*0.005*	2 (3.7%)	*0.082*
Calprotectin ≥ 10000 μg/g, yes (%)	10 (5.6%)	7 (6.8%)	*0.412*	0 (0%)	*0.277*	2 (50%)	< *0.001*	1 (1.85%)	*0.153*
Abdominal computed tomography									
Ileum inflammation, yes (%)	29 (16.2%)	20 (19.4%)	–	1 (5.6%)	–	3 (75%)	–	5 (9.3%)	–
Caecum inflammation, yes (%)	88 (49.2%)	67 (65.1%)	–	3 (16.7%))	–	3 (75%)	–	15 (27.8%)	–
Right colon inflammation, yes (%)	92 (51.4%)	71 (68.9%)	–	3 (16.7%)	–	2 (50%)	–	16 (29.6%)	–
Transverse colon inflammation, yes (%)	79 (44.1%)	61 (59.2%)	–	3 (16.7%)	–	2 (50%)	–	13 (24.1%)	–
Left colon inflammation, yes (%)	98 (54.8%)	55 (53.4%)	–	14 (77.8%)	–	3 (75%)	–	26 (48.2%)	–
Sigmoid colon inflammation, yes (%)	92 (51.4%)	52 (50.5%)	–	13 (72.2%)	–	2 (50%)	–	25 (46.3%)	–
Pancolitis, yes (%)	46 (25.7%)	37 (35.9%)	< *0.001*	2 (11.1%)	*0.135*	2 (50%)	*0.261*	5 (9.3%)	*0.001*

*NSAID* non-steroidal anti-inflammatory drugs.

*When compared to the rest of the cohort.

### Presentation of patients with acute colitis

#### Clinical presentation

One hundred sixty-nine (94.4%) patients described having experienced abdominal pain, 92 (51.4%) reported at least one episode of fever and 147 (82.1%) suffered from diarrhea (Table 1). On admission, 35 patients (19.8%) had a fever, 46 (25.7%) presented tachycardia (heart rate > 100/min) and one (0.6%) had a mean arterial blood pressure < 60 mmHg. Abdominal pain elicited by palpation (tenderness) was present in 167 patients (93.3%) and was diffuse in 26 patients (14.3%). Abdominal guarding was found in 44 patients (24.6%) and was diffuse in 1 patient (0.6%). Twenty-six patients (14.5%) had rebound pain (Table 1).

#### Laboratory results

On admission, four patients (2.2%) had anemia (defined as hemoglobin < 100 g/l). Sixty-two patients (34.6%) had elevated white blood cells (> 12G/l), 105 (58.7%) had a CRP value ≥ 50 mg/l and 22 (12.3%) a CRP value ≥ 200 mg/l. Fecal calprotectin was ≥ 250 μg/g in 132 patients (73.7%) (Table 1).

#### Computed tomography

**Figure 1:**
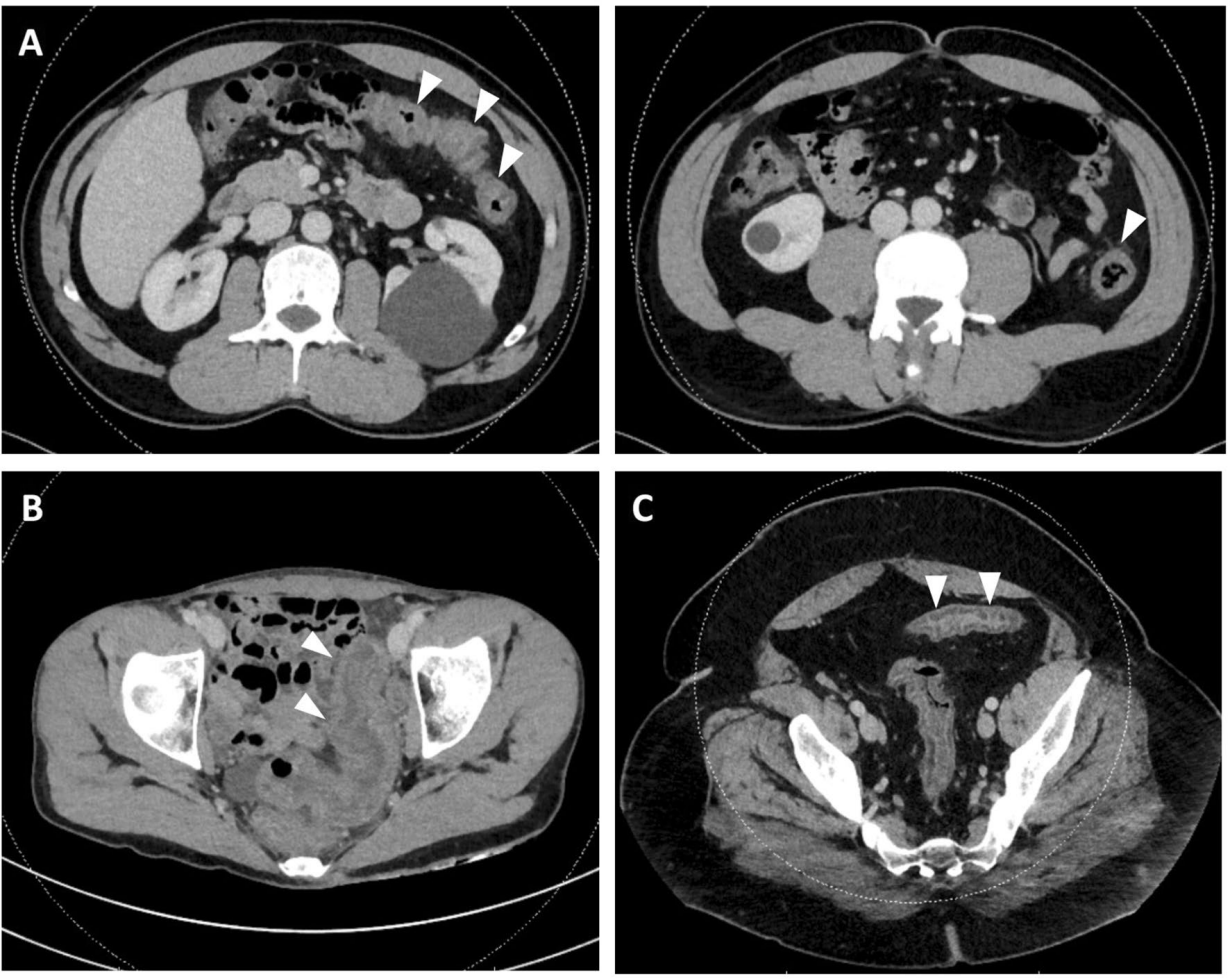
Computed tomography of episode of acute colitis. Arrows indicate areas of colonic inflammation with colonic wall thickening and infiltration of pericolonic fat. (**A1** + **2**) 46 yo male patient with infectious colitis due to *Campylobacter jejuni*. (**B**) 31 yo female patient with undetermined colitis. (**C**) 42 yo male patient with infectious colitis due to *Shigella sonei*.

On CT, 29 patients (16.2%) had inflammation of the terminal ileum, 88 (49.2%) of the caecum, 92 (51.4%) of the ascending colon, 79 (44.1%) of the transverse colon, 98 (54.8%) of the descending colon and 92 (51.4%) of the sigmoid colon. Forty-six patients (25.7%) had diffuse inflammation of the colon (pancolitis) (Table 1, Fig. 1). One patient with positive FilmArray GI panel for *Entamoeba histolytica* had a 5.5 cm abscess of segment VIII of the liver.

### Diagnostic tests looking for the etiologies of acute colitis

#### BD-Max and FilmArray GI panel

##### BD-Max

Routine PCR targets were *Campylobacter* spp.,* Salmonella* spp., *Shigella* spp.,* EHEC and EIEC* in all patients, whereas *Clostridioides difficile* and *Yersinia* spp. were searched in 143 (79.9%) and 11 (6.1%) patients, respectively (Table 2). Other pathogens were searched in 7 patients by prescribing physician. Routine PCR was positive in 93 patients (52%). Identified pathogens were *Campylobacter* spp. (54 patients, 30.2%), *Clostridioides difficile* (17 patients, 9.5%), *Salmonella* spp. (9 patients, 5%), *Shigella* spp. (6 patients, 3.4%), *EHEC* (5 patients, 2.8%), *EIEC* (2 patients, 2.2%) and *Yersinia* spp. (1 patient, 0.6%) (Table 2).

**Table 2:** Microbiological examination of the feces.

	BioFire FilmArray GI panel				Routine PCR			Culture	
	Detectable pathogens	Tested, n (%)	Positive, n (%)	In association, n (%)	Detectable pathogens	Tested, n (%)	Positive, n (%)	Detectable pathogens	n (%)
Bacteria	*Campylobacter jejuni, coli, upsaliensis*	179 (100%)	56 (31.3%)	11 (19.6%)	*Campylobacter* spp.	179 (100%)	54 (30.2%)	*Campylobacter jejuni*	45 (83.3%)
	*Clostridioides difficile (toxin A/B)*	179 (100%)	21 (11.7%)	4 (19%)				*Campylobacter coli*	8 (14.8%)
	*Plesiomonas shigelloides*	179 (100%)	1 (0.6%)	1 (100%)	*Clostridioides difficile*	143 (79.9%)	17 (9.5%)	–	–
	*Salmonella*	179 (100%)	8 (4.5%)	1 (12.5%)	*Salmonella* spp.	179 (100%)	9 (5%)	*Salmonella enteritidis*	1 (11.1%)
	*Yersinia enterolitica*	179 (100%)	2 (1.1%)	2 (100%)				*Salmonella group B*	3 (33.3%)
	*Vibrio parahaemolyticus, vulnificus, cholerae*	179 (100%)	1 (0.6%)	0 (0%)				*Salmonella group C*	2 (22.2%)
	*Vibrio cholerae*	179 (100%)	0 (0%)	0 (0%)				*Salmonella group D*	1 (11.1%)
	*Enteroaggregative E. coli (EAEC)*	179 (100%)	6 (3.4%)	2 (33.3%)				*Salmonella* spp.	2 (22.2%)
	*Enteropathogenic E. coli (EPEC)*	179 (100%)	13 (7.3%)	9 (69.2%)	*Yersinia* spp.	11 (6.1%)	1 (0.6%)	*Yersinia enterocolitica*	1 (100%)
	*Enterotoxigenic E. coli (ETEC)*	179 (100%)	3 (1.7%)	3 (100%)	*Shigella* spp.	179 (100%)	6 (3.4%)	*Shigella sonei*	3 (50%)
	*Shiga-like toxin-producing E. coli (STEC/EHEC)*	179 (100%)	5 (2.8%)	2 (40%)				*Shigella flexneri*	3 (50%)
	*Escherichia coli 0 :157*	179 (100%)	0 (0%)	0 (0%)	*EHEC*	179 (100%)	5 (2.8%)	*EHEC*	1 (20%)
	*Shigella/enteroinvasive E. coli (EIEC)*	179 (100%)	8 (4.5%)	3 (37.5%)	*EIEC*	179 (100%)	2 (2.2%)	–	–
Parasites	*Cryptosporidium* spp.	179 (100%)	0 (0%)	0 (0%)					
	*Cyclospora cayetanensis*	179 (100%)	0 (0%)	0 (0%)					
	*Entamoeba histolytica*	179 (100%)	2 (1.1%)	1 (50%)					
	*Giardia lamblia*	179 (100%)	0 (0%)	0 (0%)					
Viruses	*Adenovirus F40/41*	179 (100%)	0 (0%)	0 (0%)					
	*Astrovirus*	179 (100%)	0 (0%)	0 (0%)					
	*Norovirus GI/GII*	179 (100%)	4 (2.2%)	4 (100%)					
	*Rotavirus A*	179 (100%)	2 (1.1%)	1 (50%)					
	*Sapovirus I, II, IV, V*	179 (100%)	2 (1.1%)	1 (50%)					
TOTAL (positive patients)	–	179 (100%)	108 (60.3%)	–	–	179 (100%)	93 (52%)	–	73 (40.8%)

Feces of patients with computed tomography-proven acute colitis were screened for pathogens using the multiarray PCR FilmArray GI panel and the PCR array BD-Max. If BD-Max was positive, pathogens were further characterized using fecal culture.

##### FilmArray GI panel

**Figure 2:**
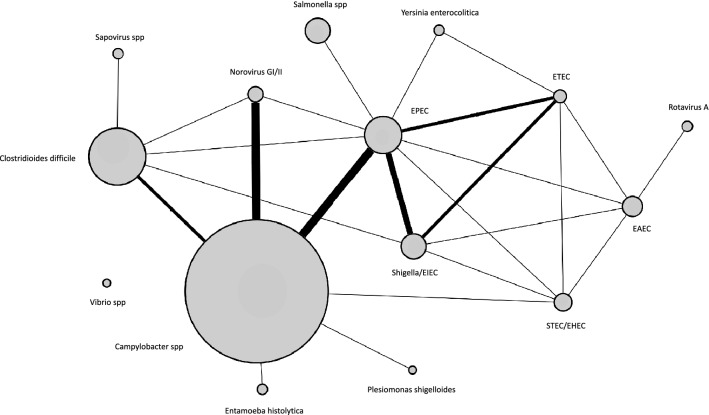
Network representation of positive targets for FilmArray GI panel. Fecal samples of patients with CT-proven acute colitis were screened using FilmArray GI panel. Every node represents a target of FilmArray GI panel, and every edge indicates whether or not this target was found in co-localization with another target in the same fecal sample. The weight of the edges indicates the number of co-localisations observed (ranging from 1 to 4), whereas the surface of the nodes indicates the prevalence of every target in the cohort.

FilmArray GI panel was performed in parallel to routine PCR in all patients and identified at least one pathogen in 108 patients (60.3%). The array was positive for *Campylobacter* spp. (56 patients, 31.3%), *Escherichia coli* spp. (27 patients, 15.1%), *Clostridioides difficile (*21 patients, 11.7%), *Salmonella* spp. (8 patients, 4.5%), *Shigella/EIEC* (8 patients, 4.5%), *Yersinia enterolitica* (2 patients, 1.1%), *Entamoeba histolytica* (2 patients, 1.1%), *Plesiomonas shigelloides* (1 patient, 0.6%), *Vibrio* spp. (1 patient, 0.6%) and viruses (8 patients, 4.5%). No patient was tested positive for *Vibrio cholerae, Escherichia coli 0157, Cryptosporidium, Cyclospora cayetanensis, Giardia lamblia, Adenovirus F 40/41* or *Astrovirus*. Some of the identified pathogens were found in association with other pathogens in the same patients (co-detection) (Fig. 2, Table 2). In patients with positive routine fecal PCR (93 patients), FilmArray GI panel was positive in 90 patients (96.8%) and negative in 3 patients (3.2%). In patients with negative routine fecal PCR (86 patients), FilmArray GI panel was positive in 18 patients (20.9%) and negative in 68 (79.1%) of patients (Fig. S3). Additional pathogens identified by FilmArray GI panel are summarized in Table S3.

#### Fecal culture

Fecal cultures were positive for *Campylobacter* spp. in 53 patients (*Campylobacter jejuni*: 45 patients (83.3%), *Campylobacter coli:* 8 patients (14.8%)), *Salmonella* spp. in 9 patients (*Salmonella group B*: 3 patients (33.3%), *Salmonella group C*: 2 patients (22.2%), *Salmonella group D*: 1 patient (11.1%), *Salmonella enteritidis:* 1 patient (11.1%), *Salmonella* spp.: 2 patients (22.2%)), *Shigella* spp. in 6 patients (*Shigella sonei:* 3 patients (50%), *Shigella flexneri*: 3 patients (50%)), *Yersinia enterocolitica* in 1 patient and EHEC in 1 (Table 2).

#### Fecal calprotectin

Fecal calprotectin was raised in all patients and had a mean value of 1922.1 ± 2895.6 μg/g. One hundred thirty-two patients (73.7%) had a calprotectin concentration ≥ 250 μg/g, 40 (22.4%) had a concentration ≥ 2500 μg/g and 29 (16.2%) had a concentration ≥ 5000 μg/g (Table 1, Fig. S4).

#### Colonoscopy

Eighty-six patients with negative BD-Max underwent colonoscopy within 6.2 ± 2.9 days after computed tomography. Colonoscopy was macroscopically and histologically compatible with ischemic colitis in 20 patients (23.3%) and with IBD in 4 patients (4.7%) (Fig. S5). Two of the patients with signs of ischemic colitis had positive samples and were therefore considered as having infectious colitis. Eight patients (9.3%) had polyps that were removed (6 patients with low grade dysplasia adenomas, 2 with hyperplastic polyps). The other patients who underwent colonoscopy (62 patients, 72.1%) had either non-specific or no inflammation of the colonic mucosa. Among them, histologically, 8 patients (12.9%) had acute inflammation, 10 had normal histology (16.1%) and 28 (45.2%) had eosinophilic infiltration. Biopsies were not performed in 16 patients (25.8%) with normal macroscopic aspect of the bowel mucosa. Fourteen patients (22.6%) with acute inflammation or no inflammation on histopathology had positive BioFire FilmArray GI panel (but otherwise negative routine PCR) (Table 3).

**Table 3:** Colonoscopy findings in patients with negative BD-Max.

Diagnosis	Patients, n (%)	Histology	Patients, n (%)	Positive FilmArray assay, n (%)	Pathogens, n
Normal or aspecific inflammation	62 (72.1%)	Inflammation	8 (12.9%)	2 (25%)	EAEC, 1*
					*Campylobacter* spp. + *Norovirus, 1*
		Eosinophilic infiltration	28 (45.2%)	7 (25%)	*Clostridioides difficile, 2*
					EPEC, 1*
					*Vibrio* spp*., 1*
					*Entamoeba histolytica, 1*
					*EAEC* + *Rotavirus, 1*
					*Yersinia* spp. + *ETEC, 1*
		No inflammation	10 (16.1%)	2 (20%)	EAEC, 2*
		No biopsy	16 (25.8%)	3 (18.8%)	EPEC, 2*
					*Clostridium difficile 1*
Ischemic colitis	20 (23.3%)			4 (20%)	*Clostridioides difficile, 1*
					*Sapovirus, 1*
					EAEC, 1*
					EPEC, 1*
Inflammatory bowel disease	4 (4.7%)	Ulcerative colitis	3 (75%)	0 (0%)	–
		Crohn’s disease	1 (25%)	0 (0%)	–
Adenocarcinoma	0 (0%)			–	–
Polyp	8 (9.3%)	Hyperplasic polyp	2 (25%)		
		Low grade adenoma	6 (75%)		
		High grade adenoma	0 (0%)		
Total	86			18 (20.9%)	

Colonoscopy was performed in patients with negative BD-Max. Biopsies were taken and sent for histological analysis.

*Considered as carriage.

#### Matching with the Swiss IBD cohort

No additional diagnosis of IBD was identified from the Swiss IBD cohort.

### Primary outcome: etiologies of computed tomography-proven acute colitis

**Figure 3:**
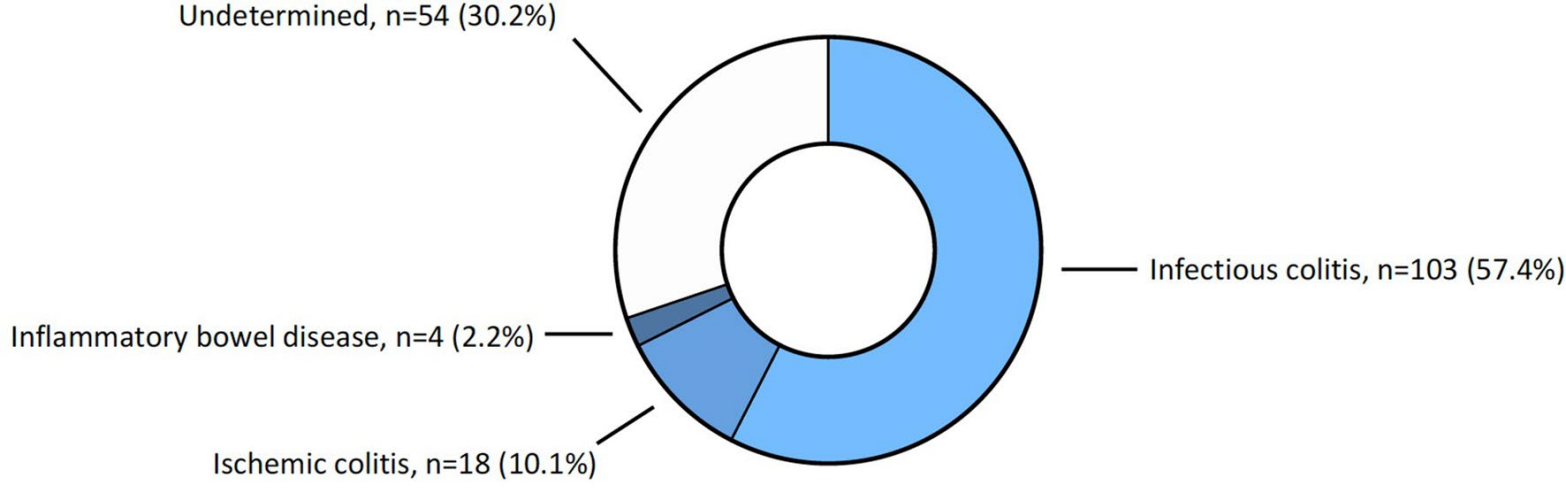
Etiologies of acute colitis were looked for using microbiological screening of the feces (BD-Max and FilmArray GI panel) and diagnostic colonoscopy in those with negative routine PCR (BD-Max).

One hundred and three (57.5%) patients had positive routine PCR and/or positive BioFire FilmArray GI panel and were therefore considered as suffering from infectious colitis. Isolated EAEC, EPEC or ETEC were considered as carriage and were therefore not considered as infectious etiology (8 patients). Eighteen patients (10.1%) with negative microbiological examination of the feces showed macroscopic and histological characteristics of ischemic colitis and were classified as suffering from ischemic colitis. Four patients (2.2%) showed characteristics of IBD on biopsies. Three were diagnosed with ulcerative colitis and one with Crohn’s disease. Finally, fifty-four patients (30.2%) had negative microbiological examination of the feces and no sign of chronic inflammation on colonoscopy and/or biopsies. Therefore, they were considered as having an episode of colitis of undetermined etiology. Of note, 43.8% of them had eosinophilic infiltration of the mucosae on histology, compatible with subacute inflammation, parasitic infection and/or iatrogenic origin (Table 4, Fig. 3). Clinical, laboratory and radiological characteristics of subgroups of patients classified by etiology are reported in Table 1.

**Table 4:** Etiologies of acute colitis.

Diagnosis	Patients, n (%)	Detailed diagnosis	Patients, n (%)*	Detailed diagnosis	Patients, n (%)*	In association, n (%)
Infectious colitis	103 (57.5%)	*Campylobacter* spp.	57 (55.3%)	*Campylobacter jejuni*	45 (43.7%)	7
				*Campylobacter coli*	8 (7.8%)	3
				*Undetermined*	4 (3.9%)	2
		*Clostridoides difficile*	23 (22.3%)	–	–	5
		*Salmonella* spp.	9 (8.7%)	*Group B*	3 (2.9%)	0
				*Group C*	2 (1.9%)	0
				*Group D*	1 (1%)	0
				*Undetermined*	2 (1.9%)	1
		*Escherichia coli* spp.	8 (7.8%)	*STEC/EHEC*	5 (4.9%)	2
				*EIEC*	2 (1.9%)	1
		*Enteroviruses*	7 (6.8%)	*Norovirus*	4 (3.9%)	4
				*Rotavirus*	2 (1.9%)	1
				*Sapovirus*	2 (1.9%)	1
		*Shigella* spp.	6 (5.8%)	*Shigella sonei*	3 (2.9%)	1
				*Shigella flexneri*	3 (2.9%)	1
		*Entamoeba histolytica*	2 (1.9%)	–	–	1
		*Yersinia enterolitica*	2 (1.9%)	–	–	2
		*Vibrio* spp.	1 (1%)	*Undetermined*	1 (1%)	0
		*Plesiomonas shigelloides*	1 (1%)	–	–	1
Ischemic colitis	18 (10.1%)	–	–	–	–	–
Inflammatory bowel disease	4 (2.2%)	Ulcerative colitis	3 (25%)	–	–	–
		Crohn’s disease	1 (75%)	–	–	–
Undetermined	54 (30.2%)	–	–	–	–	–

Etiologies of acute colitis were looked for using microbiological screening of the feces (BD-Max and FilmArray GI panel) and diagnostic colonoscopy in those with negative routine PCR (BD-Max).

*Proportion calculated using the main diagnosis as denominator.

### Secondary outcome: characteristics of patients with a final diagnosis of IBD

Patients with IBD were significantly younger than other patients diagnosed with colitis (25 ± 4.7 years vs. 53.8 ± 20.3 years, *p* = 0.005). Clinical presentation did not differ from other patients, excepted for rebound abdominal pain, which was found more often in patients with IBD (75% vs. 13.1%, *p* = 0.002). Further, anemia with haemoglobin < 100 g/l (25% vs. 1.71%, *p* = 0.002), leucocytes count > 12G/l (75% vs. 33.7%, *p* = 0.002), and fecal calprotectin values ≥ 7500 μg/g and ≥ 10,000 μg/g (50% vs. 8.57%, *p* = 0.005, and 50% vs. 4.57%, *p* < 0.001, respectively) were more often found in IBD patients. Of note, fecal calprotectin was the highest in patients with IBD when compared to patients not diagnosed with IBD (8511 ± 9438.4 μg/g, *p* < 0.001) (Table 1, Fig. S4). When analyzing only patients with negative microbiological examination of the feces, ROC curve analysis of calprotectin indicates an optimal cut-off point for diagnosis of IBD at 624.8 μg/g (sensitivity: 100%, specificity: 62%, area under the ROC curve: 0.81).

## Discussion

The medical literature is dramatically scarce regarding the definition of acute colitis and its etiologies. Numerous publications refer to the term infectious colitis to describe viral and/or bacterial episodes of diarrhea without any confirmation of colonic inflammation by imaging modalities, therefore mostly referring to episodes of gastroenteritis^9,10^. Further, most studies reporting the prevalence of bacterial pathogens during episodes of diarrhea identified low proportions of positive samples^11,12^, reflecting the low sensitivity of standard fecal cultures and the non-targeted selection of patients. To our knowledge, only one study investigated symptomatic patients admitted in emergency with CT-proven episode of colitis but suffered from information bias, with no broad screening of the feces and endoscopy reserved to a not clearly defined subgroup of patients^13^. Therefore, we thought that the recent emergence of PCR-based multi-array assays could constitute a significant advance in the field of etiological research for colitis by decreasing the number of false-negative results and guiding antibiotic therapy.

In the present study, we systematically screened the feces of patients with CT-proven acute colitis for infectious etiologies using the FilmArray GI panel, besides the routine PCR workflow (BD-Max). Out of the 179 included patients, 103 (57.5%) had positive routine PCR and/or positive FilmArray GI panel. The FilmArray GI panel allowed identifying a pathogen in 60.3% of patients, a proportion that is higher than the proportions reported in episodes of gastroenteritis detected using the same panel^14–16^, which is of 39.7% as reported by a recent systematic review and meta-analysis^17^. This higher proportion of identified pathogens might be notably explained by a more precise selection of patients in our cohort, which included symptomatic patients with radiologic evidence of colonic inflammation. Of note, studies applying better selection of patients with gastrointestinal symptoms obtained a proportion of positive samples of up to 71% using the same assay^18^. Similarly, the spectrum of pathogens encountered in the present study was different than the spectrum of pathogens identified during episodes of gastroenteritis. For instance, the prevalence of *Campylobacter* spp. in our study population was of 31.8% (representing 55% of the 58% of patients with infectious colitis). We previously published a systematic review and meta-analysis reporting on the prevalence of different pathogens in patients with gastroenteritis identified with the same multiplex PCR assay as in our cohort study, and found a prevalence of *Campylobacter* spp. of 11.8%^17^. We believe that the difference may result from a different population selection. For instance, in the present cohort study, we included only patients with symptomatic colonic inflammation demonstrated by CT, and not only patients suffering from “gastroenteritis”, which may regroup a wide variety of symptoms and clinical presentations. Moreover, we noted that several patients had co-infections with different pathogens (co-detection by molecular panel). This was previously documented in fecal samples collected in other settings, with co-infection rates ranging from 9.8 to 38%^14,15,19–21^, and sometimes detection of up to 6 pathogens^22^. This co-detection may be the result of synchronous co-infection by different pathogens or of false positives. This latest possibility is, however, less likely as previous studies have reported that the multiplex PCR assay used in our study had a specificity ≥ 97.1% when compared to fecal cultures and molecular methods^22^. Also, in our study, the majority of positive identification was confirmed by additional methods performed in parallel, as reported in Table 2.

Fecal calprotectin is a protein produced by neutrophils that accumulates in the feces in case of bowel inflammation or infection. Recent applications for calprotectin include the early diagnosis of IBD (calprotectin showed a 93% sensitivity and a 86% specificity to distinguish IBD from irritable bowel syndrome^23^) and the detection of recurrence of both Crohn’s disease and ulcerative colitis^24–26^. Similarly, calprotectin concentration is increased during bacterial diarrhea^27^ or colorectal cancer. In our study, we showed that fecal calprotectin was elevated in patients suffering from CT-proven colitis with a mean concentration of 1922.1 ± 2895.6 μg/g, therefore showing the important colonic mucosal inflammation in these patients.

Further, we performed colonoscopy in patients with negative routine PCR panel examination (BD-Max) of the feces (accounting for 48% of the cohort). Colonoscopy allowed diagnosing ischemic colitis in 10.1% of included patients, and IBD in 2.2% (three patients with ulcerative colitis and one with Crohn’s disease). No colorectal cancer was found. Moreover, in our cohort, 30.2% of included patients had negative microbiological examination of the feces and no sign of chronic inflammation on colonoscopy and/or biopsies, and were considered as having an episode of colitis of undetermined etiology. Calprotectin concentration was significantly lower in these patients than in the rest of the cohort, but still, 50% of patients with an undetermined episode had a calprotectin concentration equal to or superior to 250 μg/g, which demonstrates an active inflammation of the gastrointestinal tract. Of note, 45.2% of them had eosinophilic infiltration of the mucosae on histology. We think these findings are compatible with a subacute inflammation, explaining the absence of positive microbiological examination of the feces, false-negative result of the microbiological examination of the feces (which is unlikely as several methods of identification were performed in parallel), infection by a pathogen not detected by the methods of identification performed in the present study or, alternatively, iatrogenic origin (for example, NSAID consumption).

We believe that patients suffering from acute colitis should be guided to the appropriate diagnostic and therapeutic cares, with the objective to generate savings by shortening hospital stays and by improving prescription of additional tests, including colonoscopy. We therefore think that an adequate and early patient stratification has important medical and economical values in this setting. Therefore, considering that patients suffering from IBD require specialized treatment and follow-up, we aimed at identifying patients suffering from IBD to better target the subpopulation of patients needing diagnostic endoscopy. First, introduction of a PCR assay with more targets allowed to better identifying patients with infectious etiology. For instance, FilmArray GI panel was positive in 20.9% of patients with otherwise negative routine PCR (BD-Max). Pathogens identified were mostly pathogens not looked for by routine PCR and/or culture. Second, we have introduced fecal calprotectin, which is currently used for the early diagnosis of IBD allowing to distinguish them from functional disorders^23^ and the detection of recurrence^24–26^. We found that calprotectin was elevated in patients with episodes of infectious colitis(as previously reported in patients with bacterial diarrhea^27^), but also in patients with ischemic colitis and undetermined colitis. The highest values were, however, reported in patients with a first episode of IBD manifesting as an episode of colitis. We then determined the optimal cut-off value allowing identifying patients with IBD and determined that value to be of 625 μg/g. Applying that value as a cut-off in our cohort would have allowed reducing the number of potentially unnecessary colonoscopy from 82 to 29, therefore resulting in a decrease of 64.6% in the number of colonoscopies.

The present study has the following strengths: (1) its prospective design reduced selection bias and missing patients otherwise eligible for inclusion, (2) the introduction of thorough screening methods for microbiological examination of the feces applied for the first time in patients with acute colitis and performed in parallel, (3) reporting for the first time clinical and para-clinical presentations, as well as etiologies, of patients with computed tomography-proven episode of colitis. Its limitations are the following: (1) the number of drop-outs (24.8%) due to protocol violation, mostly due to absence of sampling, but which was in the range of the proportion planned in our preliminary sample size calculation, (2) the absence of colonoscopy in patients with positive microbiological examination of the feces, which could have led to miss potential cases of IBD and/or colorectal cancer. On this latest aspect, however, cross-matching of our cohort with the Swiss IBD cohort did not identify additional cases of IBD. And finally (3) potential patients’ selection as some patients with CT-proven acute colitis may have been treated as outpatients if not referred to the recruiting hospital.

## Conclusion

CT-proven colitis was mostly of infectious etiology (57.4%). The main pathogens identified were *Campylobacter* spp. (55.3%), *Clostridioides difficile* (22.3%) and *Salmonella* spp. (8.7%). Ischemic colitis (10.1%) and IBD (2.2%) were seldom represented. No colorectal cancer was found.

## Supplementary Information

Supplementary Figure S1.

Supplementary Figure S2.

Supplementary Figure S3.

Supplementary Figure S4.

Supplementary Figure S5.

Supplementary Table S1.

Supplementary Table S2.

Supplementary Table S3.

## Abbreviations

CT: Computed tomography
EAEC: Enteroaggregative *Escherichia coli*
EHEC: Enterohaemorragic *Escherichia coli*
EIEC: Enteroinvasive *Escherichia coli*
EPEC: Enteropathogenic *Escherichia coli*
ETEC: Enterotoxigenic *Escherichia coli*
IBD: Inflammatory bowel disease
NSAID: Non steroidal anti-inflammatory drug
ROC: Receiver operating characteristics
STEC: Shiga-like toxigenic *Escherichia coli*
