# Supplementary figures and images for: Mapping of etiologies of computed tomography-proven acute colitis: a prospective cohort study
Source: Sci Rep. 2022 Jun 13;12:9730. doi: 10.1038/s41598-022-13868-w (PMC9192641; doi:10.1038/s41598-022-13868-w)

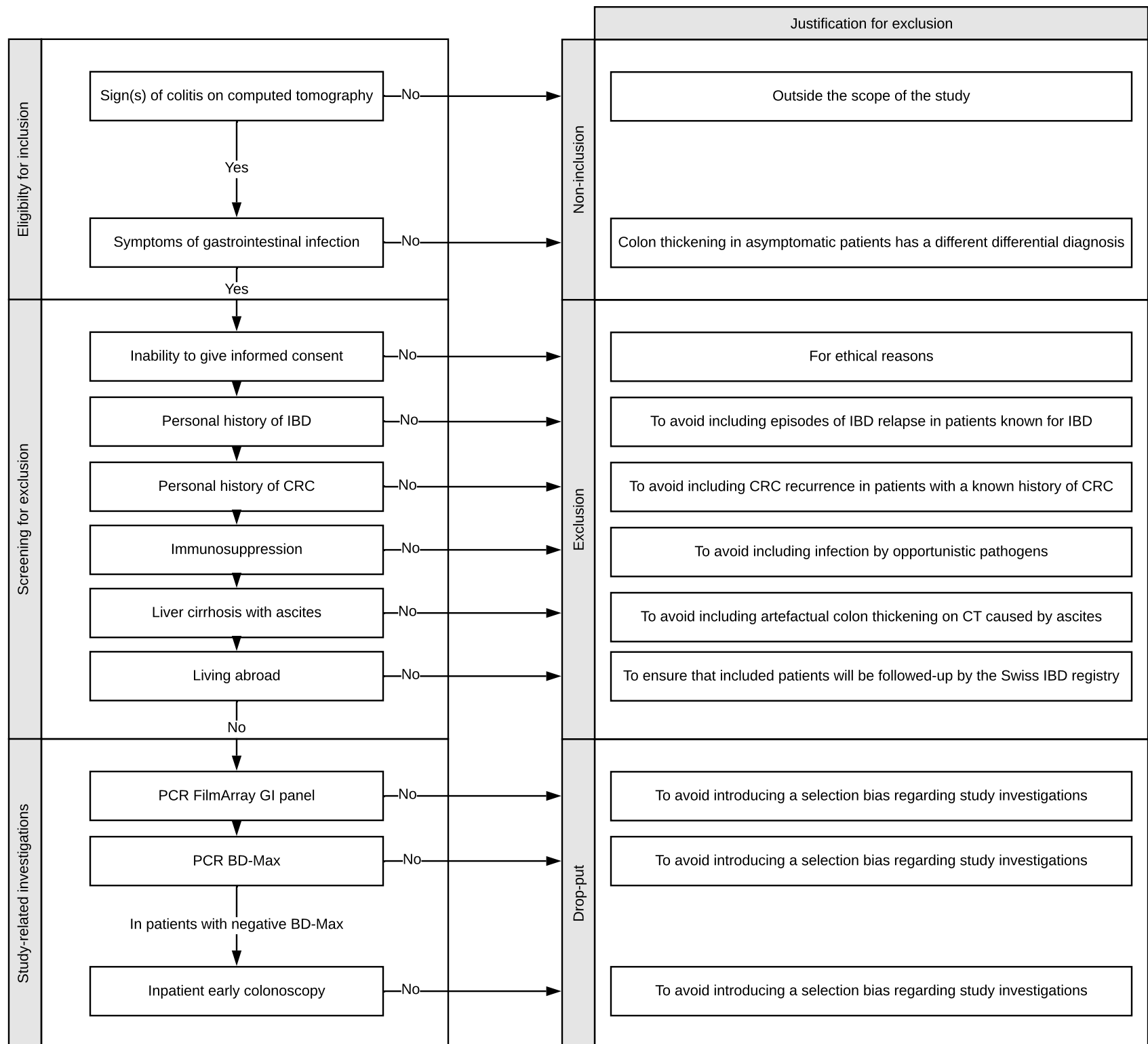

Supplement: Supplementary file 1 — Supplementary Figure S1. [file 41598_2022_13868_MOESM1_ESM.pdf]

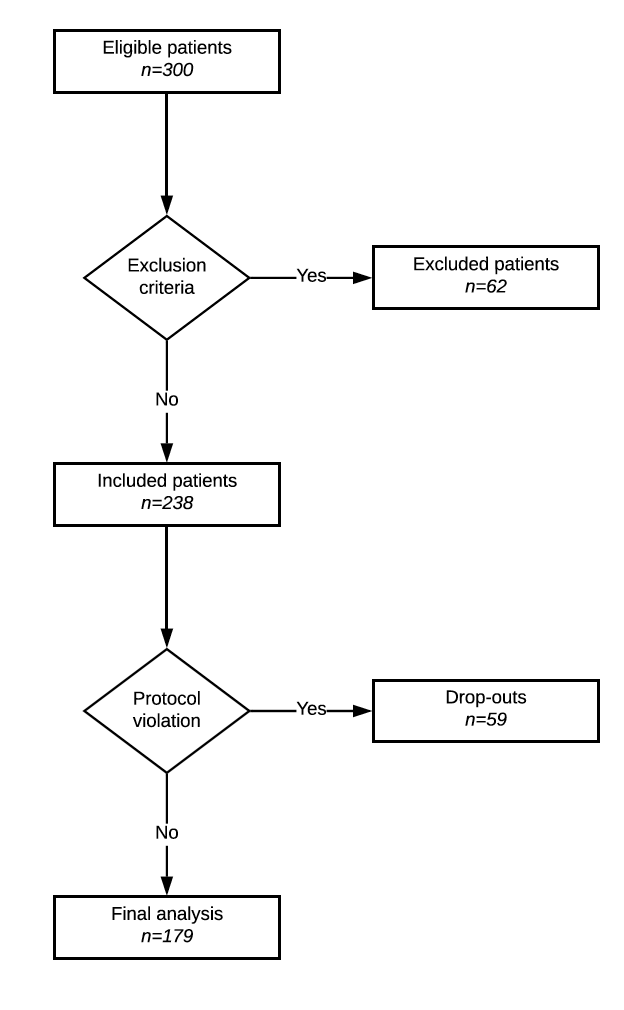

Supplement: Supplementary file 2 — Supplementary Figure S2. [file 41598_2022_13868_MOESM2_ESM.png]

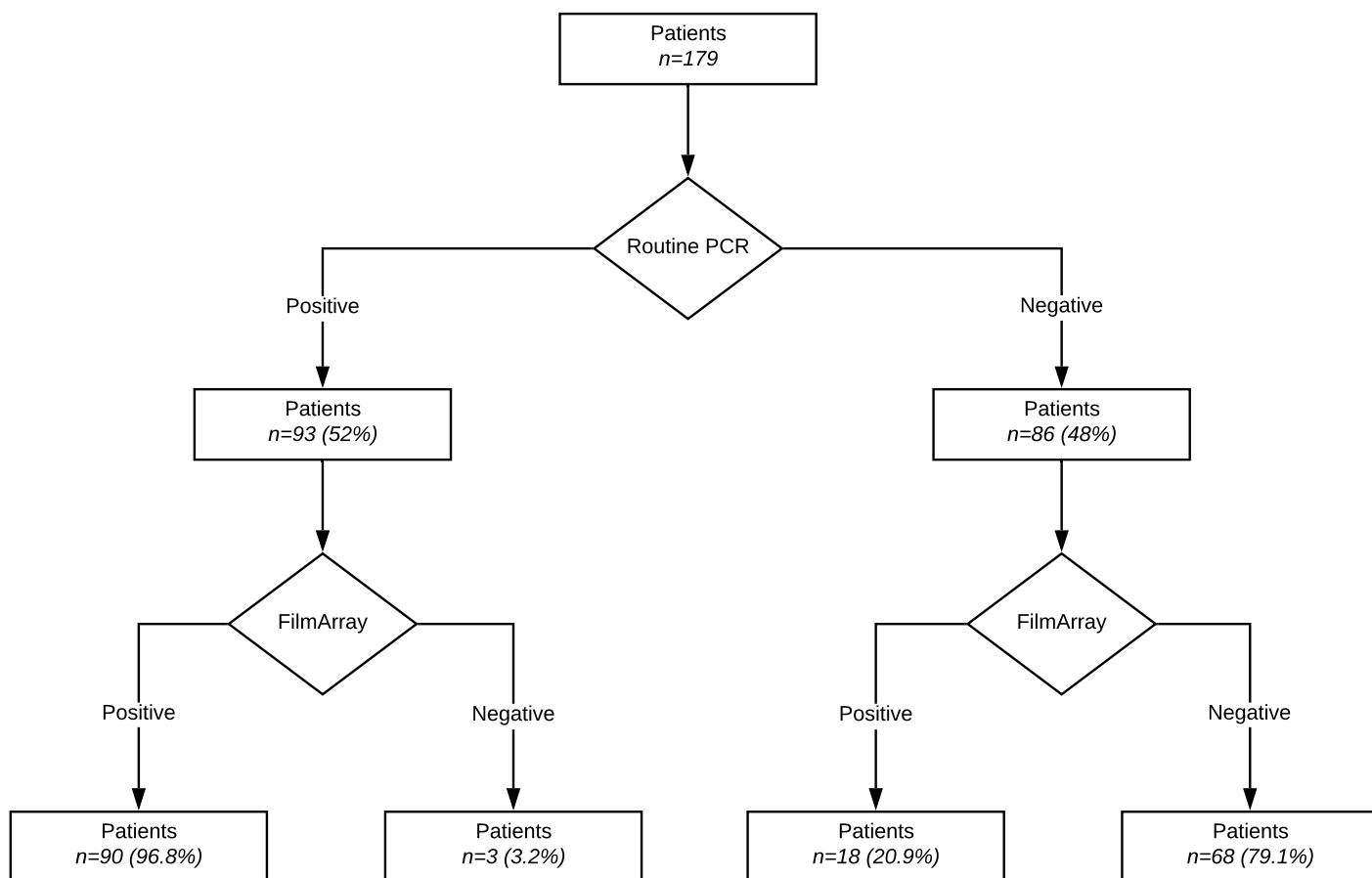

Supplement: Supplementary file 3 — Supplementary Figure S3. [file 41598_2022_13868_MOESM3_ESM.pdf]

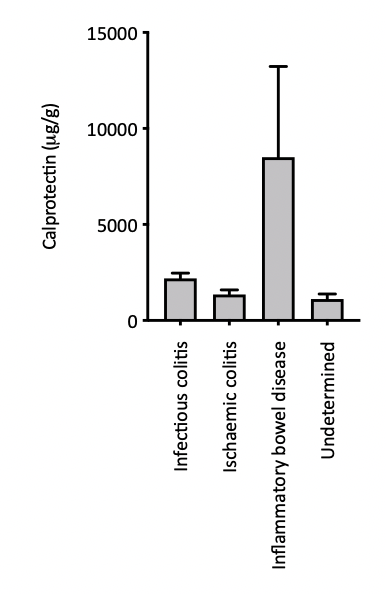

Supplement: Supplementary file 4 — Supplementary Figure S4. [file 41598_2022_13868_MOESM4_ESM.png]

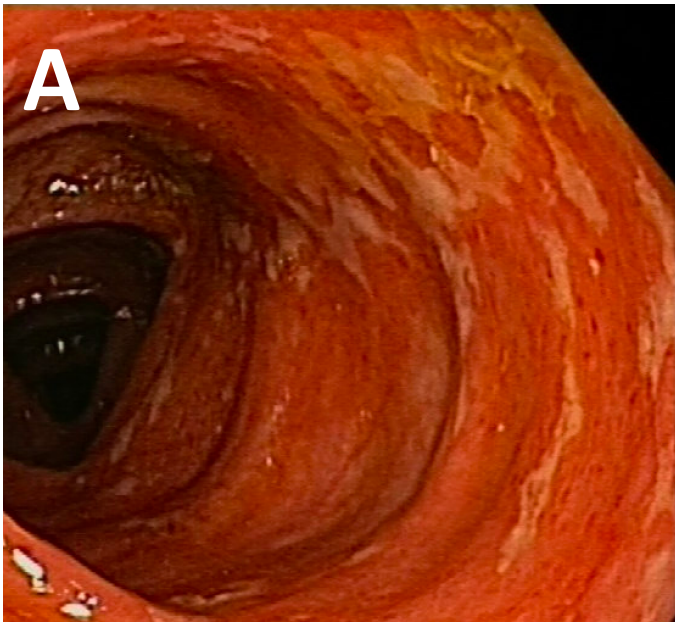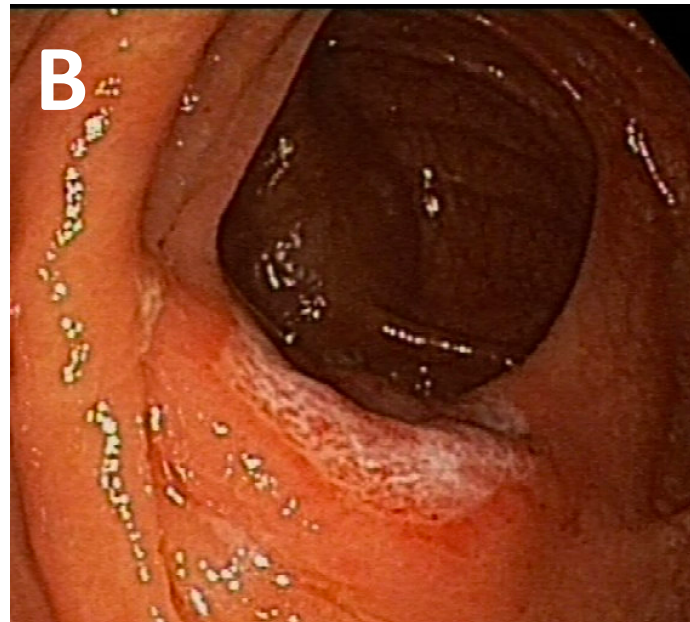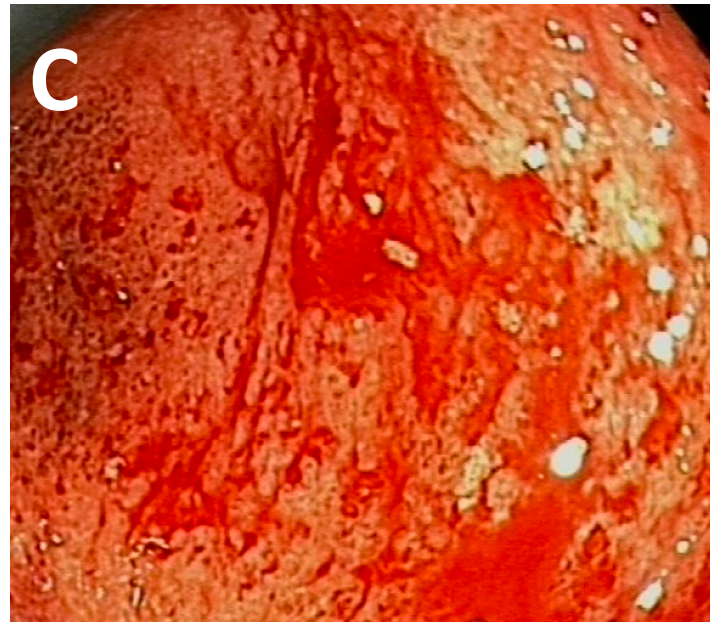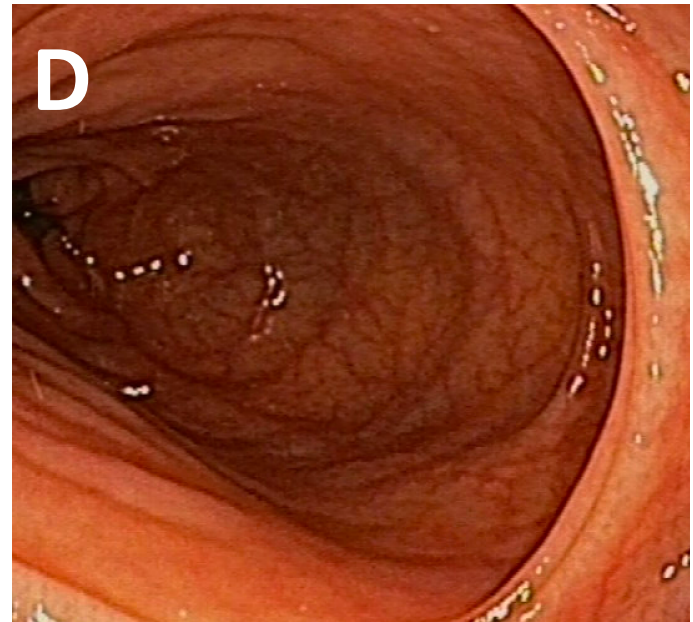

Supplement: Supplementary file 5 — Supplementary Figure S5. [file 41598_2022_13868_MOESM5_ESM.pdf]
