# Supplementary material for: Mapping of etiologies of computed tomography-proven acute colitis: a prospective cohort study
Source: Sci Rep. 2022 Jun 13;12:9730. doi: 10.1038/s41598-022-13868-w (PMC9192641; doi:10.1038/s41598-022-13868-w)
Supplement: Supplementary file 6 — Supplementary Table S1. [file 41598_2022_13868_MOESM6_ESM.docx]

|  | **Population** | **Intervention** | **Control** | **Outcome** |
| --- | --- | --- | --- | --- |
| Primary objective | Patients suffering from acute colitis | FilmArray, BD-Max, colonoscopy | - | Identification of aetiologies |
| Secondary objective | Patients suffering from acute colitis | FilmArray, calprotectin, colonoscopy | - | Identification of IBD patients |
