# Supplementary material for: Mapping of etiologies of computed tomography-proven acute colitis: a prospective cohort study
Source: Sci Rep. 2022 Jun 13;12:9730. doi: 10.1038/s41598-022-13868-w (PMC9192641; doi:10.1038/s41598-022-13868-w)
Supplement: Supplementary file 8 — Supplementary Table S3. [file 41598_2022_13868_MOESM8_ESM.docx]

| **Patient n°** | **Additional pathogens identified by FilmArray GI panel** |
| --- | --- |
| 1 | *Clostridioides difficile* |
| 2 | *EPEC* |
| 3 | *Clostridioides difficile* |
| 4 | *EAEC* |
| 5 | *Campylobacter spp + Norovirus* |
| 6 | *EPEC* |
| 7 | *EAEC* |
| 8 | *EAEC* |
| 9 | *Sapovirus* |
| 10 | *Clostridioides difficile* |
| 11 | *Yersinia enterocolitica + ETEC* |
| 12 | *EPEC* |
| 13 | *Clostridioides difficile* |
| 14 | *EAEC + Rotavirus* |
| 15 | *Vibrio spp* |
| 16 | *EAEC* |
| 17 | *EPEC* |
| 18 | *Entamoeba histolytica* |
